# Prevalence and Risk Factors of Teenage Pregnancy at Burji District, Southwestern Ethiopia; Mixed Approach Study

**DOI:** 10.3389/ijph.2025.1608389

**Published:** 2025-05-23

**Authors:** Samuel Dessu Sifer, Abatalem Minlargeh Abere, Belete Worku

**Affiliations:** ^1^ Department of Epidemiology and Biostatistics, School of Public Health, Yekatit 12 Hospital Medical College, Addis Ababa, Ethiopia; ^2^ College of Public Health and Medical Sciences, Jimma University, Jimma, Ethiopia; ^3^ Department of Public Health, Yanet College, Addis Ababa, Ethiopia

**Keywords:** teenage pregnancy, associated factors, Burji district, Ethiopia, risk factors

## Abstract

**Objectives:**

In Africa, the overall prevalence of adolescent pregnancy is 18.8%, with the sub-Saharan African region having a rate of 19.3%. The highest rates are found in East Africa at 21.5%, and in Ethiopia, the prevalence is even higher at 23.6%.

**Methods:**

A community-based cross-sectional explanatory sequential mixed approach study was carried out among 458 teenagers in Burji District from 15 January to 15 February 2024. A multistage systematic sampling technique for quantitative and purposive sampling for qualitative data was used. The quantitative data were collected through face to face interview using a structured and pretested questionnaire by trained data collectors while qualitative data were gathered through in depth interviews and focus group discussions. Variables with p-value less than 0.05 were declared as statistically significant.

**Results:**

The prevalence of teenage pregnancy was 21.8% (95% CI: 18.0, 26.0). Factors associated with teenage pregnancy included rural residence (AOR: 3.68; 95%CI: 1.29, 10.44), being married (AOR: 2.75; 95%CI: 1.06, 7.15), not using contraceptives (AOR: 5.75; 95%CI: 2.22, 14.86), and not discussing reproductive health issues (AOR: 2.60; 95%CI: 1.04, 6.76). In addition, the qualitative study identified lack of education and access to resources, social and relationship influences, cultural influences and economic and future outlook as a common cause of teenage pregnancy.

**Conclusions:**

Consequently, there is a high prevalence of teenage pregnancy in the area. Furthermore, place of residence, marital status, contraceptive use, and discussions about reproductive health issues were identified as independent factors associated with teenage pregnancy.

## Introduction

Teenage pregnancy refers to the pregnancy of adolescent girls aged 13–19 years [1]. It is a widespread public health issue and a significant concern for women’s reproductive health globally, affecting both developed and developing countries [2]. Globally, around 150 out of every 1,000 births are to adolescent girls aged 15–18 [3].

Teenage pregnancy is more prevalent in the developing world with lower Gross Domestic Product (GDP) [4]. In Africa, the overall prevalence of adolescent pregnancy is 18.8%, while in the sub-Saharan African region; it is 19.3% [5]. The highest rates are found in East Africa at 21.5%, and in Ethiopia, the prevalence is even higher at 23.6% [5, 6].

Around 90% of teenage pregnancies in the developing world occur among married girls [7]. This is often due to societal expectations that view pregnancy as a desirable outcome of marriage, pressuring young brides to have children soon after marriage [8]. In contrast, in developed regions, teenage parents are typically unmarried, and adolescent pregnancy is regarded as a social issue [7, 8].

The age at which a woman becomes pregnant can significantly impact her future health and that of her child [7]. Early childbearing carries multiple consequences, affecting maternal health, child health, and the overall wellbeing of society [2]. Adolescent girls often struggle to attend class and complete their education after marriage and pregnancy, particularly when they are under 18 years old [3]. Pregnancy among adolescents presents both medical and public health challenges, negatively impacting the social and physical development of the mother and affecting the reproductive quality of the community [4].

Several sources have identified factors contributing to teenage pregnancy, such as not residing with parents, low socioeconomic status, early sexual activity, and insufficient knowledge about contraception [4–7]. In Ethiopia, there is limited information on the prevalence and determinants of teenage pregnancy, with a notable absence of data specific to the study area.

Burji District was selected for this study due to its high vulnerability to teenage pregnancy, influenced by a combination of socioeconomic, cultural, and healthcare access challenges. The district is characterized by limited access to reproductive health services, lower educational attainment, and deeply rooted traditional practices, which may contribute to the persistence of teenage pregnancy. Additionally, existing reports and anecdotal evidence suggest that teenage pregnancy rates in Burji are higher than in neighboring areas, necessitating a focused investigation to inform targeted interventions and policy responses. Therefore, this study aimed to assess the prevalence of teenage pregnancy and the factors associated with it among teenagers in Burji District, South Ethiopian Region, Southern Ethiopia, in 2024.

## Methods

### Study Design, Area and Period

This study was carried out among female teenagers in Burji District from January 15 to 15 February 2024. Burji is situated 539 km south of Addis Ababa, with Soyama serving as the administrative center. According to a 2022 population estimate, the district has a total population of 80,495, with 39,443 men and 41,052 women. Of these women, 10,352 are teenage girls. The district includes 24 rural kebeles and 2 urban kebeles. Burji District is served by five health centers, twenty-four health posts, and one primary hospital [8].

A community based cross sectional explanatory sequential mixed methods study was carried out. A mixed-methods approach was necessary for this study because it allows for a comprehensive understanding of the prevalence of teenage pregnancy and its associated factors in Burji District by integrating both quantitative and qualitative data. By combining both methods, this study ensures statistical validity through quantitative analysis while also uncovering deeper contextual meanings through qualitative inquiry. This integration enhances the depth, accuracy, and applicability of findings, making it possible to not only identify key determinants of teenage pregnancy but also understand how and why they influence adolescent reproductive health outcomes.

### Population

The source population for the quantitative study consisted of all female teenagers aged 13–19 years in Burji District while the qualitative study involved all purposively selected teenagers in Burji district. The study population comprised all registered teenagers in the selected kebeles within this age group. The study included female teenagers aged 13–19 who had been residing in the selected kebeles for at least 6 months. Those who were severely ill and unable to communicate were excluded from the study.

### Sample Size Determination and Procedure

The sample size for this quantitative study was calculated using a single population formula with the following assumptions: a standard normal distribution corresponding to a significance level of α = 0.05 (The Z-value (1.96) corresponds to a 95% confidence interval (CI), meaning there is a 5% probability of committing a Type I error), a margin of error of 5% (the acceptable level of deviation from the true population parameter) at a 95% confidence interval, a teenage pregnancy prevalence of 23.6% from a previous study conducted in area similar population [9], a design effect of 1.5 (to account for the intra-cluster correlation and participants are selected within specific geographic areas (villages), and an additional 10% to account for non-response. This resulted in a required sample size of 458. For the qualitative component, twenty individuals took part in focus group discussions, and twelve in-depth interviews were conducted across two groups. The qualitative data collection was ceased when idea saturation is ensured. The principle of saturation was followed, continuing the sampling process until no new information or insights emerged, ensuring a comprehensive examination of the topic.

A multistage sampling technique was employed in the study. The process involved the following steps:

1. Stratification: All kebeles in Burji District were categorized into urban and rural areas.
2. Sampling of Kebeles: A simple random sampling method was used to select one out of the two urban kebeles and seven out of the 24 rural kebeles.
3. Census and Registration: A census was carried out in the selected kebeles to register all teenagers and prepare a sampling frame. In households with more than one teenage female, one was randomly selected using a lottery method.
4. Proportional Allocation: The total number of teenagers in the selected kebeles was 3,563, and the study population was proportionally allocated to each kebele.
5. Systematic Random Sampling: To finalize the study participants, systematic random sampling was conducted with an interval of 8 (Kth) in each kebele, resulting in a total of 458 participants

For the qualitative part of the study, participants were selected using a purposive sampling technique. The qualitative data were translated, transcribed, and coded through qualitative analysis methods. The insights gained from the qualitative component were then triangulated with the quantitative findings where appropriate.

### Variables

The study focused on teenage pregnancy as the dependent variable. Independent variables assessed included marriage, contraceptive nonuse, age, age at marriage, early sexual activity, sexual abuse, educational status, parents’ educational status, family communication with teenagers about reproductive health issues, parents’ marital status, residence, and family income.

### Operational Definitions

Communication of family with teenagers on RH issues: parents who discussed at least one of RH issues (menstruation, contraceptive, love, friendship, STD, sexual intercourse) [1].

Sex at an early age: having had first sexual intercourse at or before the age of 14 years [2].

Contraceptive nonuse: sexually active teenagers who did not report using any contraceptive method during the interview.

Sexual abuse: any action that pressures someone into unwanted sexual activity.

### Data Collection Procedures and Quality Assurance

Quantitative data were assembled by a structured, interviewer-administered questionnaire that had been pre-tested for reliability. The tool was adapted from the WHO’s illustrative questionnaire for youth interview surveys, with necessary modifications made to suit the study context. Initially developed in English, it was translated into Amharic by a language specialist and subsequently back-translated to English to verify consistency and clarity. Data collection was conducted by two BSc-level nurses and one BSc midwife, all under the supervision of a trained health officer. To maintain data quality, both the supervisor and data collectors underwent prior training, and a pretest was conducted on 5% of the sample population in a separate area to refine the tool. Throughout the data collection period, the supervisor and principal investigator manually reviewed questionnaires for completeness. Additionally, qualitative transcripts were retranslated into English to support accurate analysis and interpretation.

In the qualitative component of the study, data collection was conducted using focus group discussions (FGDs) and in-depth interviews (IDIs). Information was thoroughly documented through both note-taking and audio recordings to ensure comprehensive capture of participants’ input. All sessions were held in the local language, and the transcripts were subsequently translated into English for analysis. To promote comfort and openness among participants, gender-matched moderators were assigned—female moderators led discussions with women, while male moderators facilitated sessions with men. This arrangement helped create an environment in which participants could freely share their views and experiences.

During the qualitative phase, transcripts from focus group discussions and in-depth interviews were processed with great care. Skilled and certified professionals handled both transcription and translation. Two independent transcribers reviewed the audio recordings and documented participants’ responses word-for-word. Any inconsistencies between the recordings and transcripts were carefully examined using member checks, and differences were resolved accordingly. The finalized transcripts were then translated into English to prepare for analysis.

To ensure the integrity of the study, essential trustworthiness criteria—including credibility, dependability, confirmability, and transferability—were thoroughly addressed. The meticulous process of transcription and translation was designed to maintain data accuracy and consistency, thereby enhancing the reliability and overall validity of the qualitative findings.

### Data Management and Data Analysis

The collected data were initially coded, cleaned, and entered using EpiData version 3.0.1, and subsequently exported to SPSS version 25.0 for statistical analysis. Descriptive analyses were conducted and the results were displayed through narrative descriptions and tables. Associations between the dependent and independent variables were examined using chi-square tests, along with bivariate and multivariable logistic regression analyses. Variables with a p-value less than 0.25 in the bivariate analysis were included in the multivariable model. Additionally, variables with a p-value greater than 0.25 but supported by biological plausibility or prior literature were also considered. A p-value below 0.05 in the multivariable logistic regression was taken to indicate statistical significance. To evaluate the influence of missing data, a sensitivity analysis was carried out. Findings were reported in both textual and tabular formats.

During the qualitative component of the study, data management was carried out with careful attention. The process began with the systematic organization of materials related to data collection, transcription, and translation to facilitate a phenomenological analysis. The translated transcripts were then comprehensively appraised numerous times to fully grasp their underlying meaning. To strengthen the study’s validity and provide deeper insights, qualitative findings were integrated with quantitative data through triangulation when applicable. This mixed-methods approach offered a more holistic perspective on the research topic. The qualitative results were ultimately grouped into four main thematic areas: economic and future aspirations, cultural norms, social and interpersonal dynamics, and barriers related to education and resource accessibility.

## Results

### Socio-Demographic Characteristics

A total of 458 teenagers participated in the study, achieving a 100% response rate. The mean age of the study participants was 16 ± 2 years. More than three-fourths respondents (357, 77.9%) were rural residents and over one-fifth of the respondents (107, 23.4%) had been married. Majority of the study participants (286, 62.5%) completed primary education (Table 1).

**TABLE 1:** Socio-demographic characteristics of teenagers for the study on prevalence and risk factors of Teenage pregnancy at Burji District, Southwestern Ethiopia; 2024.

Variables	Category	Frequency (n)	Percentage (%)
Age (years)	13–14	130	28.4
	15–17	153	33.4
	18–19	175	38.2
Residence	Urban	101	22.1
	Rural	357	77.9
Educational status of the respondent	No formal education	62	13.5
	Primary education	286	62.5
	Secondary and above	110	24
The marital status of the respondent	Unmarried	351	76.6
	Ever married	107	23.4
Age at marriage (n = 107)	<18	30	28
	≥18	77	72
Mothers’ educational status	No formal education	97	21.2
	Primary education	317	69.2
	Secondary and above	44	9.6
Father’s educational status (n = 446)	No formal education	62	13.9
	Primary education	297	66.6
	Secondary and above	87	19.5
Annual family income (ETB)	<18,000	37	8
	18,000–90,000	292	63.8
	>90,000	129	28.2
Parents marital status	Married and living together	400	87.3
	Separated and divorced	40	8.7
	Widowed	18	4

### Causes of Teenage Pregnancy

#### Lack of Education and Access to Resources

Information about sexual health, lack of school-based education on safe sex practices, poor access to contraceptives in rural areas, and feelings of embarrassment when seeking reproductive health services were common barriers identified by participants.

A study participant from FGD reported that:- “*… I don’t have enough information about sex and contraception*”. Similarly, another study participant from in depth interview stated that:- “*… my school doesn’t teach me about safe sex practices.*” A study participant from FGD stated that:- “*… since I am living in a rural area, it’s hard to get birth control pills or condoms in our area*”. Moreover another study participant mentioned that, “*… I was too embarrassed to go to the clinic to get contraception*”.

#### Economic and Future Outlook

Economic hardship, limited educational and career opportunities, and a lack of future prospects were major factors that influenced participants’ decisions toward early pregnancy.

A study participant from in depth interview reported as: “*… I thought having a baby would give me some financial support from the government*”. Similarly, another study participant from FGD mentioned that: “*… I didn't see any other future for myself, given our financial situation.*” A study participant from FGD replied as: - “*… I didn’t think I’d have a chance to go to college or get a good job anyway.*” Moreover, a study participant from FGD stated that: - “*… with no real career prospects, having a baby seemed like a good idea.*”

#### Cultural Influences

A study participant from FGD reported that: - “*… in our culture, getting married and having kids early is common*”. Similarly, another study participant from FGD stated that: - “*…there’s a lot of pressure from family and society to prove your fertility*”. Additionally, a study participant from in depth interview stated that: - “*… my parents never talked to me about sex or birth control*”. Moreover, a study participant from in depth interview stated that: - “*…coming from a large family, it seemed normal to have kids young.*”

### Sexual and Reproductive Health Related Influences

Among respondents who live with both or either of their parents, 140 (30.7%) respondents had not discussed their parents on the reproductive health issue. 163 (35.6%) respondents had experienced sexual intercourse, and out of those sexually active respondents, 26 (16%) of them initiated sex at an early age (Table 2).

**TABLE 2:** Sexual and reproductive health characteristics of teenagers for the study on prevalence and risk factors of Teenage pregnancy at Burji District, Southwestern Ethiopia; 2024.

Variables	Category	Frequency (n)	Percentage (%)
Sexual intercourse	Yes	163	35.6
	No	295	64.4
Age at first initiation of sex (n = 163)	≤14	26	16
	>14	137	84
Contraceptive use (n = 163)	Yes	89	54.6
	No	74	45.4
Sexual abuse	Yes	13	2.8
	No	445	97.2
Discussion on RH issue (n = 456)	Yes	316	69.3
	No	140	30.7

A study participant from an in depth interview was reported as: - “*… my friends were all having sex, and I felt pressured to do it too.*” Similarly, another study participant from FGD mentioned that: - “*… everyone around me is dating and having sex; it feels like it’s what you’re supposed to do*”. A study participant from FGD stated as: - “*… my boyfriend didn’t want to use protection*”. Moreover, a study participant from in depth interview reported as: - “*… I thought having a baby would keep my partner with me.*” A study participant from FGD stated that: - “*… we were drinking and it just happened*”. Similarly, another study participant from in depth interview stated that: - “*… when you’re high or drunk, you don’t think about protection.*”

### Prevalence of Teenage Pregnancy

The prevalence of teenage pregnancy among respondents in Burji District was 21.8% (95% CI: 18, 26). Among them, 14 (14%) were currently pregnant.

#### Factors Associated With Teenage Pregnancy

In the bivariate analysis, variables with a p-value less than 0.25—including residence, marital status, annual estimated family income, discussion on reproductive health issues, contraceptive use, and the respondent’s educational status—were considered candidates for multivariable logistic regression. Respondent’s educational status was included in the final model based on biological plausibility, while marital status was retained in consideration of findings from previous studies.

This analysis revealed significant associations between teenage pregnancy and factors such as residence, marital status, discussion on RH issues, and contraceptive use. Teenagers living in rural areas were 3.7 times more likely to become pregnant compared to their urban counterparts (AOR: 3.68; 95% CI: 1.29, 10.44). Those who were ever married had higher odds of pregnancy compared to unmarried teenagers (AOR: 2.75; 95% CI: 1.06, 7.15). Additionally, not using contraceptives increased the likelihood of teenage pregnancy by 5.75 times compared to contraceptive users (AOR: 5.75; 95% CI: 2.22, 14.86). Teenagers whose parents did not discuss RH issues with them had more than twice the risk of pregnancy compared to those whose parents did (AOR: 2.60; 95% CI: 1.01, 6.76) (Table 3).

**TABLE 3:** Factors associated with teenage pregnancy for the study on prevalence and risk factors of Teenage pregnancy at Burji District, Southwestern Ethiopia; 2024.

Variables	Category	Teenage pregnancy		COR (95% CI)	AOR (95% CI)
		Yes	No		
Residence	Urban	11	90	1	
	Rural	89	268	2.72 (1.39, 5.31)^a^	3.68 (1.29, 10.44)^b^
Marital status	Unmarried	21	330	1	1
	Ever married	79	28	37.62 (23.93, 82.15)^a^	2.753 (1.06, 7.154)^b^
Annual family income (birr)	<18,000	18	19	12.63 (4.96, 32.18)^a^	3.65 (0.84, 15.91)
	18,000–90,000	73	219	4.44 (2.15, 9.19)^a^	11.97 (0.21, 71.41)
	>90,000	9	120	1	1
Educational status of the respondent	No formal education	20	42	0.77 (0.400, 1.486)^a^	1.55 (0.86, 2.77)
	Primary education	121	165	1.19 (0.756, 1.863)^a^	1.31 (0.68 (2.53)
	Secondary and above	42	68	1	1
The marital status of the respondent	Unmarried	36	315	0.280 (0.163, 0.482)^b^	0.80 (0.52, 1.25)
	Ever married	31	76	1	1
Discussion on the RH issue	Yes	49	267	1	1
	No	51	89	3.12 (1.97, 4.94)^a^	2.60 (1.01, 6.76)^b^
Contraceptive use	Yes	39	50	1	1
	No	61	13	6.02 (2.89, 12.49)^a^	5.75 (2.22, 14.86)^b^
Educational status	No formal education	18	44	0.78 (0.66, 2.53)^a^	0.36 (0.06, 2.01
	Primary education	44	242	0.34 (1.75, 4.82)^a^	0.51 (0.17, 1.49)
	Secondary and above	38	72	1	1

^a^ Indicates variables with p-value <0.25 in bivariate analysis and.

^b^ Indicates variables with p-value <0.05 in multivariable analysis.

## Discussion

Teenage pregnancy remains a major public health issue worldwide, with an estimated 16 million teenage girls giving birth annually in developing countries [3]. These make them at increased risk of experiencing pregnancy-related complications such as preeclampsia, gestational hypertension, premature birth, low birth weight, and an increased likelihood of cesarean delivery [4]. Teen mothers are also at a higher risk of experiencing postpartum depression, anemia, and inadequate prenatal care, which can impact both their health and the health of their infants [3, 4].

In this study, the prevalence of teenage pregnancy was found to be 21.8% (95% CI: 18, 26). This finding is consistent with a study conducted as Columbia (21%) [5], Sub Saharan Africa (24.88%) [6], Kenya (18.1%) [7] and North West Ethiopia (25.4% [8]. This similarity could be attributed to related study designs, cultural backgrounds, and socio-demographic factors. However, the prevalence found in this study is lower compared to a study conducted in Bangladesh (35%) [9], Pakistan (42%) [10] and East Africa (54.6%) [11]. Among the nationwide studies, it is lower than Northeast Ethiopia (28.6%) [12] and eastern Ethiopia (30.2%) [13]. This difference can be attributed to variations in study design, sample size, and study area. On the other hand, the prevalence found in this study is higher than that reported by Getachew Mulu Kassa, which was 10.1% [14]. This discrepancy could be attributed to differences in the outcome variable, as the other study measures the prevalence of childbearing rather than including all pregnancies.

Place of residence was significantly associated with teenage pregnancy. Teenagers from rural areas were 3.7 times more likely to be pregnant than those from urban areas. This finding aligns with studies conducted in the northern part of Ethiopia [8, 12]. This might be related to the limited access to healthcare services in rural areas, including reproductive health education and family planning resources [5]. This lack of access may increase the likelihood of teenage pregnancy, as young girls may not receive adequate information or services to prevent unintended pregnancies. This might also be related to, fewer role models who are pursuing education or careers in rural areas, leading them to follow traditional paths that include early marriage and pregnancy. Policymakers and public health practitioners should prioritize expanding access to reproductive health services and education in rural areas, while also promoting girls’ education and empowerment initiatives to address the higher risk of teenage pregnancy associated with rural residence.

Marital status was found to be a factor associated with teenage pregnancy. Married teenagers were more than twice as likely to become pregnant as their unmarried counterparts. Many studies conducted in East Africa, Sub Saharan Africa and Ethiopia support this finding [11, 13–18]. This is particularly evident in contexts where early marriage is common, as it often leads to early childbearing. This highlights that early marriage increases the risk of teenage pregnancy due to a lack of awareness about contraception and reproductive health [19]. In addition, teenage girls in marital unions often face social pressures and limited opportunities for education, which can exacerbate the risk of unintended pregnancies [20]. Moreover, this may be due to the fact that adolescent girls who are married are more likely to engage in frequent and unprotected sexual activity, which often results in early and high-risk first births. In many developing countries, it has been shown that fertility commonly occurs within the first year of marriage [9]. Additionally, the influence of husbands in discouraging contraceptive use may also contribute to this outcome [10]. The findings of this study highlight the importance of investing in efforts to end early marriage as a key strategy for reducing teenage pregnancy and its associated health complications.

Contraceptive nonuse is identified as a factor significantly associated with teenage pregnancy. Consistent with this finding, studies conducted in Sub Saharan Africa [6], Uganda [21] and Ethiopia [8, 12, 17], contraceptive non-users were more likely to become pregnant than contraceptive users. This might be related to lack comprehensive knowledge about reproductive health and access to accurate information on modern contraceptive methods, cultural taboos and social stigma surrounding premarital sexual activity can discourage teenagers from seeking contraceptive services, even when they are sexually active [6]. Additionally, limited availability of youth friendly reproductive health services, fear of being judged by healthcare providers, and misconceptions about side effects can further hinder contraceptive uptake. In some cases, power imbalances in relationships or lack of negotiation skills may prevent young girls from making decisions about contraceptive use [4, 6, 20]. These factors collectively contribute to the increased risk of unintended pregnancies among teenagers who do not use contraception. Policymakers and public health practitioners should implement multi-sectoral strategies to prevent teenage pregnancy by enforcing laws that prohibit early marriage, promoting girls’ education and empowerment, expanding access to comprehensive sexual and reproductive health education, and ensuring the availability of youth-friendly contraceptive services that are culturally sensitive and non-judgmental.

Lack of education and access to essential resources has been widely acknowledged as a major causes of teenage pregnancy. Adolescents who have limited educational attainment are less likely to receive comprehensive information about sexual and reproductive health, which increases their vulnerability to early and unintended pregnancies. Education plays a crucial role in delaying the onset of sexual activity, promoting contraceptive use, and empowering young girls to make informed decisions about their reproductive health [11]. Furthermore, lack of access to youth-friendly health services, family planning resources, and accurate reproductive health information disproportionately affects adolescents in rural and low-income settings, contributing significantly to high teenage pregnancy rates [12]. Similarly, when adolescents are deprived of education and essential support services, they are more likely to engage in risky sexual behaviors due to ignorance or misinformation [13]. These findings suggest that improving access to education and reproductive health resources is vital in the prevention of teenage pregnancy. Policymakers and public health practitioners should prioritize expanding access to quality education and youth-friendly reproductive health services—particularly in rural and underserved areas—to empower adolescents with the knowledge and resources needed to prevent early and unintended pregnancies.

Social and relationship influences have been extensively explored as contributing factors to teenage pregnancy. Peer pressure, family dynamics, and romantic relationships often play a significant role in shaping adolescents’ sexual behavior. Teenagers who experience poor parental communication or lack emotional support from family are more likely to engage in early sexual activity, increasing their risk of pregnancy [22]. Additionally, peer influence, especially within groups where early sexual activity is normalized, can lead adolescents to make risky decisions without adequate knowledge or protection [23]. Addressing these influences through youth empowerment, relationship education, and family engagement is therefore essential in reducing teenage pregnancy rates.

Cultural influences and economic outlook have been acknowledged as important underlying causes of teenage pregnancy, particularly in low- and middle-income countries. In many societies, cultural norms that promote early marriage and childbearing contribute significantly to high rates of adolescent pregnancies. In communities where early marriage is customary, girls often face pressure to prove fertility soon after marriage, leading to early pregnancies [24]. Moreover, highlighted that traditional gender roles, stigma around contraceptive use, and the expectation of motherhood at a young age reinforce the likelihood of teenage pregnancy [25].

From an economic perspective, poverty is a key driver, as economically disadvantaged adolescents may have limited access to education, health services, and employment opportunities. This lack of economic security often results in early sexual initiation, transactional sex, or early marriage as a survival strategy, thereby increasing the risk of pregnancy. Consistently, girls from low-income households are more likely to become pregnant during adolescence due to limited life choices and aspirations [26]. These findings underscore the need for culturally sensitive interventions and poverty-reduction strategies to address the root causes of teenage pregnancy.

Making open discussions between parents and teenagers about reproductive health (RH) issues was noted as factor associated with teenage pregnancy. Teenagers whose parents did not discuss RH issues with them were 2.6 times more likely to become pregnant compared to those whose parents had these discussions. This finding aligns with studies conducted in Ethiopia [14, 18]. This might be related to lack of communication on RH matters often leaves adolescents uninformed about safe sexual practices, the consequences of early pregnancy, and available contraceptive options [27]. Cultural taboos, embarrassment, and lack of knowledge among parents often hinder such discussions, further increasing adolescents’ vulnerability [15]. Therefore, promoting parent-teen communication about reproductive health is a critical strategy in efforts to reduce teenage pregnancy.

### Limitation of the Study

Due to the cross-sectional nature of the study design, causality could not be established. Additionally, some sensitive information may have been underreported, and calculating household income for farmers posed challenges in this study. Moreover, it has a potential for bias from the purposive sampling technique used in the qualitative component, which could influence the diversity of perspectives gathered.

### Conclusion

Teenage pregnancy remains a pressing public health concern in Burji District, influenced by a combination of socio demographic and behavioral factors. The findings underscore the critical need for targeted interventions that address rural-urban disparities, promote adolescent reproductive health education, and enhance communication between parents and teenagers on sexual and reproductive health issues. Efforts to prevent early marriage and improve access to and utilization of contraceptive methods among adolescents are also essential. Strengthening community-based strategies and youth-friendly services could play a pivotal role in mitigating the risks associated with teenage pregnancy and improving the overall wellbeing of adolescents in the area.

## Abbreviations

AOD, Adjusted Odd Ratio; CI, Confidence Interval; COD, Crude Odd Ratio; EDHS, Ethiopian Demographic and Health Survey; ETB, Ethiopian Birr; GDP, Gross Domestic Product; RH, Reproductive Health; SNNPR, Southern Nations Nationalities and Peoples Region; STD, Sexually Transmitted Disease; UNICEF, United Nations International Children’s Emergency Fund; WHO, World Health Organization.
